# G4 Matters—The Influence of G-Quadruplex Structural Elements on the Antiproliferative Properties of G-Rich Oligonucleotides

**DOI:** 10.3390/ijms22094941

**Published:** 2021-05-06

**Authors:** Carolina Roxo, Weronika Kotkowiak, Anna Pasternak

**Affiliations:** Department of Nucleic Acids Bioengineering, Institute of Bioorganic Chemistry, Polish Academy of Sciences, Noskowskiego 12/14, 61-704 Poznan, Poland; croxo@ibch.poznan.pl

**Keywords:** G-quadruplex, UV melting, circular dichroism, antiproliferative activity, anticancer agents

## Abstract

G-quadruplexes (G4s) are non-canonical structures formed by guanine-rich sequences of DNA or RNA that have attracted increased attention as anticancer agents. This systematic study aimed to investigate the anticancer potential of five G4-forming, sequence-related DNA molecules in terms of their thermodynamic and structural properties, biostability and cellular uptake. The antiproliferative studies revealed that less thermodynamically stable G4s with three G-tetrads in the core and longer loops are more predisposed to effectively inhibit cancer cell growth. By contrast, highly structured G4s with an extended core containing four G-tetrads and longer loops are characterized by more efficient cellular uptake and improved biostability. Various analyses have indicated that the G4 structural elements are intrinsic to the biological activity of these molecules. Importantly, the structural requirements are different for efficient cancer cell line inhibition and favorable G4 cellular uptake. Thus, the ultimate antiproliferative potential of G4s is a net result of the specific balance among the structural features that are favorable for efficient uptake and those that increase the inhibitory activity of the studied molecules. Understanding the G4 structural features and their role in the biological activity of G-rich molecules might facilitate the development of novel, more potent G4-based therapeutics with unprecedented anticancer properties.

## 1. Introduction

Guanosine-rich oligonucleotides (GROs) can fold into four-stranded structures named G-quadruplexes (G4s) in the presence of monovalent cations such as K^+^ or Na^+^ [1,2,3]. G-quadruplexes are non-canonical nucleic acid structures characterized by the stacking of two or more successive planes of four guanine residues, named G-tetrads, in a nearly planar arrangement by interacting via Hoogsteen hydrogen bonding. The G-quadruplex (G4) structure is highly polymorphic and can be formed by one, two or four strands of DNA or RNA. The folding topology is dependent on the molecularity of the structure, orientation of the strands in the core and position or composition of the loops [1].

Recently, several putative G-quadruplex-forming sequences were found in RNA and DNA fragments naturally occurring in living organisms. Importantly, such G-quadruplex triggers are perceived as key regulatory elements in pivotal cell processes, such as replication, transcription, translation and genome instability, and are often found in the promoter regions of cancer-related genes [4,5]. Consequently, G-quadruplex structures constitute an attractive target in gene regulation therapeutic strategies. However, synthetic G-rich oligonucleotides that form G-quadruplex structures have been demonstrated to be a promising therapeutic tool. Such molecules can recognize different proteins and inactivate their biological functions. G-quadruplexes have several advantages compared with unstructured sequences, such as single-stranded DNA or RNA oligonucleotides, e.g., higher thermodynamic and chemical stability, improved cellular uptake, versatile chemical modification and low immunogenicity. G-quadruplexes have been extensively studied in recent years, and their various targets, such as cancer cells [6,7,8,9], viruses [10,11,12] and proteins [13,14], were revealed. In particular, G-rich oligonucleotides are perceived as cancer-selective antiproliferative agents [15,16,17]. One of the most studied anticancer aptamers and the most clinically advanced G-quadruplex is AS1411, first discovered by Bates et al. [18]. This G-quadruplex aptamer has attracted much attention and expectation in anticancer therapy because it demonstrates antiproliferative activity in many cell lines, such as breast, cervical and prostate cancer cell lines [18]. The biological activity of AS1411 is related to its binding to nucleolin, a protein involved in cell survival, growth and proliferation [19]. Surface nucleolin is mainly overexpressed on the membrane of cancer cells. A high level of this protein is associated with increased cell proliferation, malignant transformation and progression, making the overexpression of surface nucleolin an indicator of a poor clinical prognosis [18]. Similarly, the antiproliferative properties of other GROs are often attributed to their specific interactions with cell-surface nucleolin. Nevertheless, up till now only few reports have explained the role of specific structural requirements of GROs, which can assure the high anticancer potential of this type of molecules. Presently, the most challenging and undoubtedly essential aim in G-quadruplex-based antitumor therapy is understanding the role of specific G-quadruplex structures, such as their diversity of folding topologies, loop length, number of G-tetrads or thermodynamic stability in the efficient inhibition of cancer cell proliferation. Such information is crucial to accurately develop and improve the anticancer properties of GROs via chemical modifications in a predictable manner.

Herein, we report a systematic study on the thermodynamic and structural properties of a series of intermolecular G-quadruplexes, as well as their therapeutic potential. The G-quadruplexes presented in this article were selected from an initial pool of twenty DNA sequences described in previous structural studies as forming G-quadruplex structures. In order to identify a correlation between the characteristic structural elements of G-quadruplexes with the antiproliferative activity of oligonucleotides, we analyzed five sequence-related DNA molecules that varied slightly in the loop length or number of G-tetrads within the core of the formed structure. Only such group of closely related sequences assure in-depth analysis of subtle changes in the loop and G-quadruplex core that influence the therapeutic potential of GROs. The structure of these G-quadruplexes was revealed to be intrinsic to their biological effect, demonstrating a complicated structure–activity relationship. The obtained information may be helpful to develop new, potent G-quadruplex-based therapeutic agents with predictable anticancer properties.

## 2. Results and Discussion

The generalization of the structural features of G-quadruplexes is intricate and challenging because even short oligonucleotides with a minor difference in sequence can differ in folding topologies, having only the G-tetrad as a conservative element. Presumably, the various folding topologies can be used by the cell machinery and might play a role in the biological activity of this type of structure in natural systems. Thus, individual structural characteristics should be considered when investigating the role of G-quadruplexes in cellular processes. Studies of correlation between G-quadruplexes′ structural elements and their biological characteristics were possible due to a carefully selected group of sequence-related oligonucleotides. We are firmly convinced that only such a narrow group of molecules, which are almost sequentially identical and maintain constant character of folding molecularity, assures reliable analysis of subtle structure­–activity relationships.

**Figure 1:**
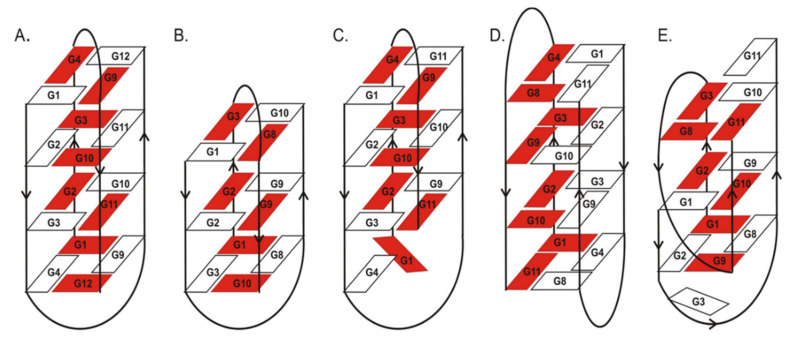
Schematic presentation of G-quadruplex structures formed by ON1 (**A**), ON2 (**B**), ON3 (**C**), ON4 (**D**) and ON5 (**E**) [22,23]. The two strands involved in the formation of each G-quadruplex structure are marked with different colors.

Previous NMR and X-ray studies described the structural features of the G-quadruplexes used in this research. The crystal and NMR structure of d(G_4_T_4_G_4_)_2_ (ON1, Figure 1A) in the presence of K^+^ ions was reported as an intermolecular G-quadruplex with four G-tetrads formed by two adjacent antiparallel strands and two diagonal loops [20]. The structure of d(G_3_T_4_G_3_)_2_ (ON2, Figure 1B) studied by NMR spectroscopy forms an antiparallel, dimeric G-quadruplex in the presence of Na^+^ or K^+^, with three G-tetrads and diagonal loops [21]. The folding topology of d(G_4_T_4_G_3_)_2_ (ON3, Figure 1C) investigated by NMR spectroscopy in the presence of Na^+^ ions consists of a bimolecular, antiparallel, diagonally looped G-quadruplex with three G-tetrads. Interestingly, this G-quadruplex structure shows asymmetry caused by two guanine residues aligned on one side of the G-quadruplex core [21]. X-ray studies of the d(G_4_T_3_G_4_)_2_ monomer structure (ON4, Figure 1D) in the presence of K^+^ ions showed an antiparallel, bimolecular G-quadruplex with four G-tetrads, lateral loops and strands arranged in the head-to-tail orientation [22]. Interestingly, the folding topology of d(G_3_T_4_G_4_)_2_ structure (ON5, Figure 1E) determined by NMR spectroscopy in K^+^ solution is completely different from the remaining closely related oligonucleotides ON1–ON4. The structure of ON5 consists of a bimolecular, asymmetric G-quadruplex with three G-tetrads and two different types of loops, i.e., diagonal and edge type. Importantly, G11 and G3 residues from one of the strands are outside the G-quadruplex core [23].

To study the physicochemical aspects of the selected G4-forming oligonucleotides, we used well-established methods. To assess the thermodynamic stability and folding topology, we applied UV melting analysis, circular dichroism (CD) spectroscopy and thermal difference spectra (TDS). As a complement, we performed biological investigations using antiproliferative studies, cellular uptake analysis, viability of oligonucleotides in human serum and their affinity to interact with nucleolin. The wide range of analyses allowed us to analyze correlations between sequence composition, G-quadruplex stability, topology and anticancer potential.

### 2.1. UV Melting Analysis

Thermodynamic studies were performed using the UV melting method, which allows the determination of detailed parameters and confirms the formation of a G-quadruplex structure at physiological temperature. Additionally, comprehensive analysis of the T_m_ dependence vs. sample concentration indicates the molecularity of folding of the studied G-quadruplex structures (Supplementary Data, Figure S1–S5). Thermodynamic analysis was performed for five 5′-FAM-labeled oligonucleotides with various loop lengths and G-content (Table 1). The oligonucleotide sequences have the potential to form G-quadruplexes with three or four G-tetrads connected in respective positions by 3- or 4-nt-long all-T type loops. The presence of a distinct dependence between T_m_ values versus various sample concentrations for all oligonucleotides confirmed that all the studied G-quadruplex structures are folded intermolecularly. One of the most influential factors in the context of the G-quadruplex thermodynamic stability is the number of G-tetrads involved in core formation. Assuming no conformational changes after initial complex formation, the number of G-tetrads should be proportional to the G-quadruplex stability [24]. Thus, it was expected that the most stable G-quadruplex structures would be formed by ON1 and ON4, which can form four G-tetrads. The structures formed by ON1 and ON4 were characterized by ΔG°_37_ values of −8.30 and −8.97 kcal/mol, respectively (Table 1). A recent report indicated that the average increase in the free energy change per G-tetrad was mostly linear, with a slope of 1.65–2.00 kcal/mol [24]. Interestingly, the presence of the fourth G-tetrad in ON1 compared with loop-isosequential ON2 caused a larger variation in the Gibbs free energy values (ΔΔG°_37_ = 2.70 kcal/mol; ΔT_M_ = 24.4 °C), likely due to the different G-quadruplex molecularity of folding and/or another folding topology observed in our studies compared with that for previously analyzed G-quadruplexes. Various structural characteristics of the molecules can contribute to the overall thermodynamic effects. Other important factors for G-quadruplex stability are the length and sequence of the loops [25]. Comparison of the Gibbs free energy values shows that the formation of the G-quadruplex with 3-nt-long loops (ON4) is more energetically favorable compared with the 4-nt-long loop (ON1). The presence of an additional thymidine in the loop destabilizes the G-quadruplex structure by 0.67 kcal/mol (ON1 vs. ON4). The literature data published so far indicate loop length preferences spanning the guanosines that are involved in G-tetrad formation depending on the specific loop types, i.e., 1- to 3-nt-long fragments needed for the lateral and double-chain reversal loop or 3- to 4-nt-long for diagonal loops [25]. Therefore, the destabilization caused by the presence of an additional thymidine residue might be caused by the less energetically favorable length of the 4-nt loop to form the specific loop type in the G-quadruplex structure formed by ON1 and ON4. Importantly, some differences in G-quadruplex folding between ON1 and ON4 were confirmed by different CD patterns (see the next section). The widths of the G-quadruplex grooves are usually dependent on specific structure topologies that differ in the distribution of negatively charged phosphate backbones. The various electrostatic forces, which must be overcome in the folding process, might constitute an additional reason for the differences in thermodynamic stability observed for ON1 and ON4 variants.

ON3 and ON5 are two oligonucleotide variants with three G-tetrads and an extra guanosine at 5′- and 3′-ends, respectively. Aromatic systems, thus also nucleosides, placed at the end of oligonucleotides can stabilize nucleic acid structures by additional stacking interactions. Indeed, the presence of an additional guanosine residue at the terminal positions of ON3 and ON5 caused increased G-quadruplex thermodynamic stability compared with ON2 (Table 1). Interestingly, the favorable energetic effect was more pronounced for ON3 with an extra G residue at the 5′-end (ΔΔG° = −2.02 kcal/mol for ON3 vs. -0.63 kcal/mol for ON5). According to Črnugelj et al., ON5 folds into an unusual, bimolecular G-quadruplex topology with two loop types (Figure 1) [23]. In one of the ON5 strands, G3 and G11 guanosines are deployed at opposite sides of the G-quadruplex core, whereas all guanosine residues in the second ON5 strand are involved in G-tetrad formation. By contrast, in ON3, the G4 residue from one strand and G1 from the other strand are not directly involved in G-tetrad formation and are aligned at the same side of the G-tetrad spanned by two diagonal loops. The lower thermodynamic stability of ON5 might be attributed to the presence of diagonal and edgewise loops that hinder G-quadruplex stacking with non-G-tetrad guanosine residues [23]. Conversely, two diagonal loops outside the core in ON3 presumably makes the stacking of G1 and G4 more favorable, leading to higher stabilization of this type of structure than the unprecedentedly folded ON5. Additionally, 5′-FAM-labeling might have serious implications on the thermodynamics of both structures due to the different folding topologies of both molecules, because the large aromatic surface of fluorescein also has various structural neighborhoods in both variants of G-quadruplexes.

To verify the influence of 5′-FAM labeling on the stability of ON1 to ON5 G-quadruplexes, the parameters for unlabeled oligonucleotides were also determined. Surprisingly, according to the T_M_ values (Table 1, italic font), the presence of fluorescein at the 5′-end of the oligonucleotides studied herein caused significant destabilization. Unlabeled oligonucleotides were characterized by higher melting temperatures with the ΔT_M_ in the range of 2.8–15.8 °C, compared with fluorescently labeled variants. Based on the above, the naked G-quadruplexes studied herein are stable at physiological temperature, which is important for the antiproliferative studies performed at 37 °C. The largest destabilization (ΔT_M_ = 15.8 °C) induced by 5′-FAM was observed for ON5 with unusual G-quadruplex topology. As mentioned previously, the positioning of the 5′-terminus of one ON5 strand in the center of the G-quadruplex core most likely makes the presence of an additional bulky fluorescent group energetically unfavorable and disrupts the interactions within G-tetrads. By contrast, the lowest destabilization was observed for ON3, which is also most likely connected with a specific structure, as already discussed. Interestingly, analysis of the ΔH° and ΔS° contribution to the change in the G-quadruplex stability indicates that, for most oligonucleotides, the unfavorable energetic effect is enthalpy driven. For ON3 only, the minor destabilization observed after oligonucleotide labeling was entropic in origin (Supplementary Data, Tables S1 vs. S2). In this case, the presence of FAM at the 5′-end of ON3 caused a favorable enthalpy change that was overbalanced by an unfavorable entropy decrease, suggesting that the positive effect of H-bonding interactions and the stacking of aromatic systems might be overcome by an unfavorable contribution from nonspecific hydrophobic interactions or the loss of rotational-translational freedom.

### 2.2. Circular Dichroism Spectra

CD spectroscopy is one of the simplest methods that can be used to characterize the G-quadruplex topology, because G-quadruplex structures of different polarities provide different CD spectral characteristics [26,27]. Although the theoretical analysis of G-quadruplex CD spectra is poorly described in the literature, some general rules to interpret G-quadruplex CD bands are established and successfully used to characterize simple G-quadruplex systems. A G-quadruplex with a parallel conformation is characterized by a CD spectrum with positive band at 260−265 nm and a negative band at 240−245 nm. By contrast, antiparallel G-quadruplex conformation typically shows a positive peak at 290−295 nm and a weaker negative band at 260−265 nm, whereas hybrid G-quadruplex conformation has positive bands at 295 and 270 nm and a negative band at 240 nm. Compared with regular DNA and RNA helix geometry, the interpretation of CD shapes of G-quadruplex structures is more complicated and ambiguous. Previously reported data have shown that although some generalizations for simple systems can be roughly made to facilitate prediction of G-quadruplex topology, they cannot be treated arbitrarily [25]. The main determinants of the CD curve shape in the case of the G-quadruplex are stacking interactions within the core, which are influenced by the rotation angle between G-tetrads. Because of the lack of detailed theoretical data analysis of the influence of looped bases and rotation between the stacks, the CD data can provide only some general conclusions about changes in G-quadruplex structure topology.

**Figure 2:**
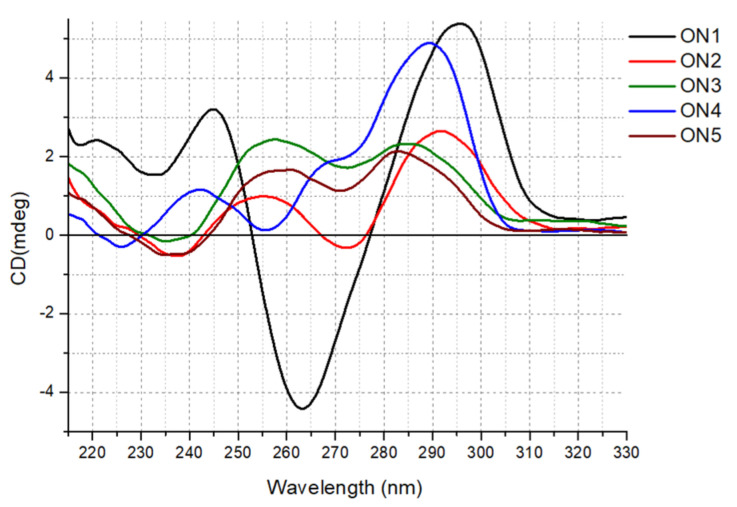
CD spectra of the unlabeled oligonucleotides ON1 to ON5 studied in buffer containing 100 mM KCl, 20 mM sodium cacodylate and 0.5 mM Na2EDTA (pH 7.0) at 37 °C.

Herein, we report for the first-time comprehensive CD analysis for unlabeled ON1–ON5. The CD spectrum obtained for ON1 at 37 °C possessed one positive band near 295 nm and one negative signal around 265 nm, indicating the formation of an antiparallel G-quadruplex structure (Figure 2). The reduction in the number of G-tetrads from four to three in ON2 dramatically changed the CD shape, resulting in a lower intensity pattern with two maxima near 290 and 255 nm and a minor minimum around 240 and 270 nm. Such reshaping of the CD curve might be due to structural polymorphism of ON2 with a predominance of antiparallel folding topology. However, according to Karsisiotis et al., antiparallel G-quadruplexes can be classified into two different groups depending on the same or distinct type of glycoside bond angle (GBA) of consecutively stacked guanosines within the core [28]. In reference to published data, the CD spectra pattern of these two groups is different as a result of substantially different GBA arrangement and various stacking of electronic transition dipole moments. Thus, the reshaping of the CD spectra of ON2 might be rather due to maintaining antiparallel topology with a changed GBA pattern of guanosines in G-tetrads. Indeed, the ON2 core contains *syn-syn-anti* and *anti-anti-syn* intrastrand stacking of guanosines, whereas ON1 is characterized by *syn-anti-syn-anti* consecutive stacking interactions [23]. Similarly, the reduction in the loop length from 4 to 3 nt in ON4 with the simultaneous retention of the number of G-tetrads compared with that in ON1 also changed the CD pattern significantly, resulting in the shift of the main, positive band from 295 nm observed for ON1 to around 288 nm for ON4. Moreover, an additional positive maximum appeared near 265 nm, indicating a mixed parallel/antiparallel topology of ON4 or, more probably, an antiparallel structure with a specific GBA arrangement of guanosines in G-tetrads.

Interestingly, two G-quadruplex structures with unpaired guanosine residues, i.e., ON3 and ON5, showed similar CD patterns with two maxima near 280 and 255 nm, as well as a minor negative band near 235 nm, which can be attributed to hybrid topology (ON5) or a specific GBA arrangement of the G-quadruplex core within the antiparallel structure with an extended higher order architecture stem, i.e., with extensive base stacking of additional guanosine residues onto the G-quadruplex core (ON3). Previously, published structural data of ON5 in 10 mM KCl indicated a topology with three strands of the G-quadruplex core aligned in parallel orientation and the fourth directed oppositely for ON5 [23]. Notably, the CD spectra of the 5′-FAM-labeled G-quadruplexes suggest that the fluorescent labeling also influences the molecular folding of the G-quadruplex structures, indicating rather hybrid or mixed topology for the studied G-quadruplexes (Supplementary Data, Figure S6).

### 2.3. Thermal Difference Spectra

Analysis of the thermal difference spectra (TDS) is often performed as a supplement to CD analysis. According to Mergny et al., the TDS of G-quadruplex structure has a specific shape, represented by two positive signals around 243 and 273 nm and a negative signal near 295 nm [29].

**Figure 3:**
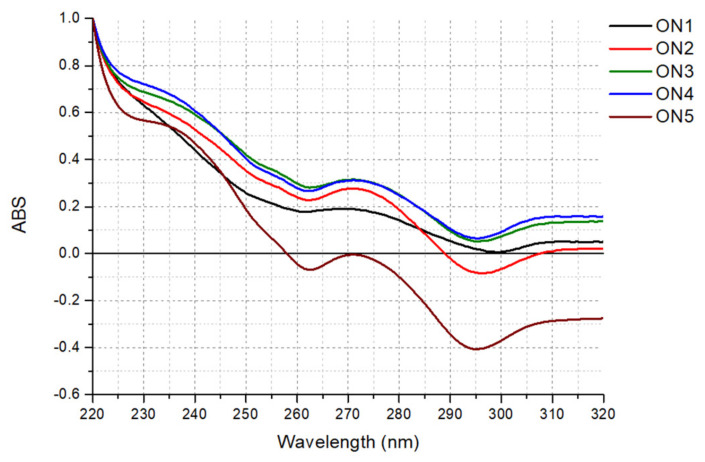
Normalized thermal difference spectra of the oligonucleotides ON1 to ON5 studied in buffer containing 100 mM KCl, 20 mM sodium cacodylate and 0.5 mM Na2EDTA (pH 7.0).

Surprisingly, the analysis of the TDS global shapes obtained for ON1 to ON5 demonstrated a deviation from the typical TDS, attributed to the G-quadruplex structure (Figure 3). Despite the similar pattern observed for all the studied oligonucleotides, the curves had only two distinct signals, i.e., a minimum around 295 nm and a maximum near 273 nm. Importantly, the TDS shapes changed after FAM labeling, revealing a third characteristic signal near 243 nm (Supplementary Data, Figure S7). Considering changes in the TDS pattern induced by the presence of the 5′-FAM label, we conclude that the results are consistent with the CD spectra and thermodynamic studies.

### 2.4. Antiproliferative Assay 

Recently, an increasing number of G-quadruplexes have been identified with well-established antiproliferative activity against human cancer cell lines—for example, AS1411 [18], modified thrombin-binding aptamer variants [30,31,32], *AT11* [33] and *G4-STAT3, G4-TOP1, G4-SP1, G4-VEGF, G4-NCL, G4-SHP-2* and *G4-TGT* [34]. Such G-rich molecules can bind to various proteins involved in many cellular pathways, particularly cell proliferation or apoptosis, and dysregulate their biological functions [35]. Simultaneously with the discovery of the potential application of G-quadruplexes as anticancer agents, the need arose to establish some general structural features of the oligonucleotides that will facilitate the development of new G-rich sequences and predict their applications for medical purposes. This goal could be achieved due to the systematic analysis of the antiproliferative activity of various G-quadruplexes in connection with their structures and thermodynamic and biological stability. Current literature data show only infrequent examples concerning the antiproliferative effect of intramolecular G-quadruplexes in connection with their serum stability [36].

**Figure 4:**
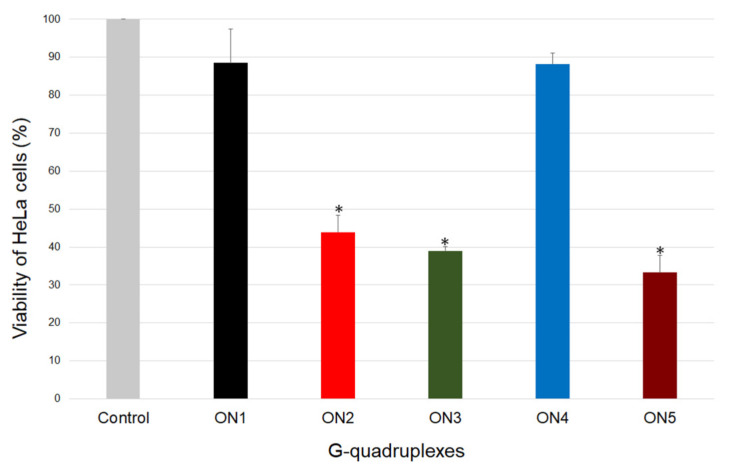
Antiproliferative activity of the oligonucleotides ON1 to ON5 studied at 10 µM. *HeLa* cells cultured without oligonucleotides constituted the control. * *p* < 0.001 by one-way ANOVA.

Herein, to evaluate the potential of intermolecular G-quadruplexes to act as a potent anticancer drug and to set a structure–activity relationship, we examined the capacity of the growth inhibition of the analyzed oligonucleotides (ON1 to ON5) in the human cervical adenocarcinoma *HeLa* cell line, using the MTT assay. This technique allows the assessment of the cell viability based on the reduction of the water-soluble, yellow tetrazole salt (MTT) into insoluble dark blue formazan [37]. The amount of reduced MTT is directly proportional to the number of living cells. The data analysis indicated that the *HeLa* cell viability was significantly reduced in the presence of ON2, ON3 and ON5 compared with the control (Figure 4, Supplementary Data, Figure S8). Furthermore, ON5 displayed the most favorable antiproliferative properties and caused a decrease in *HeLa* cell viability up to 33% after 7 days of treatment (Figure 4). The presence of ON2 and ON3 in the growth medium displayed slightly weaker but almost comparable effects (the cell viabilities were 44 and 39%, respectively; Figure 4). The common structural features of the above G-quadruplexes were the presence of three G-tetrads and longer loops containing four thymidine residues (Table 1). By contrast, the most thermodynamically stable oligonucleotides, ON1 and ON4, which possess four G-tetrads and 4- or 3-nt in loops, respectively, demonstrated no significant antiproliferative activity in *HeLa* cells (Figure 4). Based on the above data, we conclude that compounds with a less compact and shorter G-quadruplex core (three G-tetrads) exert a more favorable inhibitory effect. One of the most likely reasons for the antiproliferative activity of particular G-quadruplexes in some cancer cell lines could be the synergistic toxicity of their guanine- and thymidine-based degradation products [36,38]; therefore, less thermodynamically stable G-quadruplexes might be more predisposed to enzymatic digestion, simultaneously becoming more potent anticancer agents.

### 2.5. Stability of Oligonucleotides in Human Serum

The efficiency of oligonucleotide action in the human body is determined, among other processes, by the grade of their elimination from the bloodstream and susceptibility to nuclease digestion [39]. G-quadruplexes can represent a potent therapeutic tool, which in general is characterized by lower vulnerability to enzymatic degradation in the biological environment than linear oligonucleotides.

**Figure 5:**
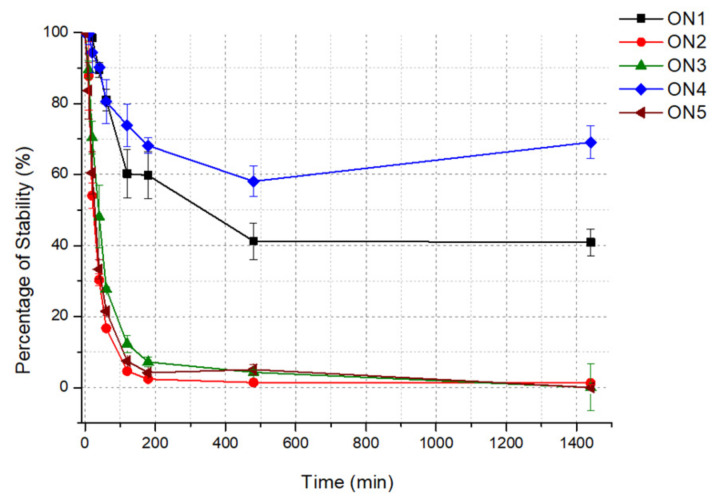
Stability of the oligonucleotides ON1 to ON5 studied in human serum at 1 pmol of G-quadruplex oligonucleotides, dissolved in 20 µl of 1× PBS containing 100 mM KCl.

Considering the above findings and to verify whether the biostability of the analyzed G-quadruplexes is in a relationship with their other physicochemical properties, we assigned the stability of the oligonucleotides in human serum. The parameter that describes the susceptibility of the oligonucleotide to nuclease digestion is the half-life (T_1/2_), defined as the time required to reduce the amount of the tested substance by half. In this study, the T_1/2_ value for all five 5′-FAM-labeled oligonucleotides was determined by incubation in human serum at 37 °C. Based on the obtained results, ON4 demonstrated the most favorable value of serum stability and almost 60% of this oligonucleotide could be detected even after 1440 min of incubation (Figure 5). A several times lower but still beneficial value of the T_1/2_ parameter was calculated for ON1 (248.03 min; Table 2). Both the above G-quadruplexes were characterized by the presence of the core formed by four G-tetrads and 3- or 4-nt-long loops, respectively. Additionally, the oligonucleotides were the most thermodynamically stable of all the analyzed compounds. By contrast, the serum stability of the G-quadruplexes possessing three G-tetrads, loops containing four thymidine residues and one additional guanosine at 5′- or 3′-terminus was significantly lower. The T_1/2_ values for these oligonucleotides were 36.64 and 35.34 min, respectively (Table 2). Noticeably decreased biostability was observed for ON2, which contains three G-tetrads and a 4-nt-long loop without an additional guanosine residue at the terminus. Furthermore, the thermal stability of this oligonucleotide was also the lowest, with the T_M_ value being relatively close to physiological body temperature.

Based on the above data, we conclude that the serum stability of the analyzed G-quadruplexes was strictly proportional to their thermodynamic properties. The more structuralized and expanded G-tetrad core and shorter loops predisposed the oligonucleotides to have extended biostability. The above correlation was presumably attributed to the fact that G-quadruplexes are highly resistant to nucleolytic cleavage, which could be possible only after G-tetrads unfolding. What is more, it has been proven that the DNase I-like endonucleases are predominantly responsible for the nucleolytic hydrolysis of DNA oligonucleotides in blood plasma [40]. The enzymes preferentially catalyze the hydrolysis of single-stranded DNA fragments; therefore, shorter loops in the G-quadruplex structure seem to have a protective influence.

### 2.6. Cellular Uptake by Flow Cytometry 

The therapeutic potential of the G-quadruplexes is determined, apart from biostability, by their ability to be taken up by the cells of interest, as well as by their cellular distribution [41]. The cellular uptake can depend on the G-quadruplex concentration, sequence and structure, and differs between various cell types. The determination of these mechanisms and subcellular distribution is essential to evaluate their therapeutic potential and mechanism of action.

**Figure 6:**
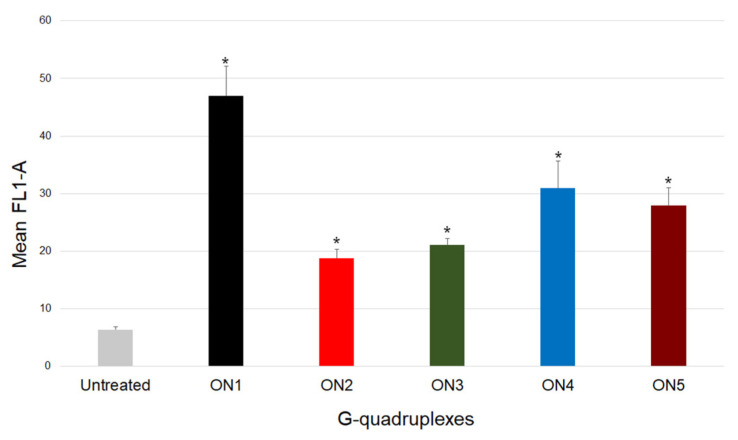
Relative cellular uptake of the oligonucleotides ON1 to ON5 studied at 10 µM in *HeLa* cells. * *p* < 0.001 by one-way ANOVA.

Herein, flow cytometry analysis was employed to estimate the cellular uptake of analyzed G-quadruplexes using 10 µM of the 5′-FAM-labeled oligonucleotides (ON1 to ON5) in *HeLa* cells. Data analysis revealed that ON1 was characterized by the highest intracellular accumulation among the five G-quadruplexes (Figure 6). Compared with the control, significant cellular uptake was also observed for ON4 and ON5. Interestingly, only the last G-quadruplex exhibited a considerable antiproliferative effect among the three variants. Additionally, compounds ON2 and ON3, which were found to have effectively restrained *HeLa* cells growth, had the lowest internalization outcome. Thus, we assumed that the efficiency of the cellular uptake is not always one of the main determinants of the antiproliferative properties of the analyzed G-quadruplexes, as it was previously proposed by Choi et al. [42].

Notably, an interesting correlation was observed between the length of the G-quadruplex and efficiency of cellular uptake. The longer oligonucleotides, which possess a higher number of G-tetrads in the core and shorter loop fragments, were characterized by more efficient internalization. The results might be explained, in part, by the axial charge density and Debye–Hückel screening phenomenon. In the situation when the charge density reaches the critical value, the system aims to neutralize this negative state through counterion condensation at the particle surface [25,43]. Moreover, it was proposed that a higher axial charge density induces the increased association of counterions to neutralize the overall charge of a molecule. Unfortunately, no data are available concerning G-quadruplexes in terms of this theory; however, some conclusions can be approximated from calculations made for the random coil oligonucleotide and the B-DNA duplex. It was observed that the latter structure has a higher axial charge density than the random coil oligonucleotide and therefore, up to 76% of its charge is neutralized, whereas for single-stranded oligonucleotides, the counterion condensation reaches only 44%. The G-quadruplex can be considered as a structural arrangement comprising a G-tetrad core and loops, corresponding to the duplex and random coil forms, respectively. Hence, oligonucleotides with a higher number of G-tetrads in the core and shorter loops possess a higher axial charge density and have a greater amount of charge neutralized, significantly facilitating their cellular internalization. The above theory, along with fact that the G-quadruplexes have higher charge density parameters than duplexes [43], can also potentially explain the well-known G-quadruplex ability to cross the cell membrane spontaneously without application of any additional carriers.

### 2.7. Ability to Bind to Nucleolin

Nucleolin (NCL) is a G-quadruplex multifunctional phosphoprotein [44] that can bind to DNA and RNA G-quadruplexes and G-rich aptamers [45]. Due to its involvement in various processes in human cells, such as ribosome biogenesis, chromatin remodeling, transcriptional regulation and apoptosis, as well as its significant overexpression in the nucleus and cytoplasm of cancer cells, it constitutes a promising target for anticancer therapy [35,46]. The examples of the inhibition of cancer cell lines through decreasing nucleolin activity via G-quadruplex binding have been reported frequently [47].

**Figure 7:**
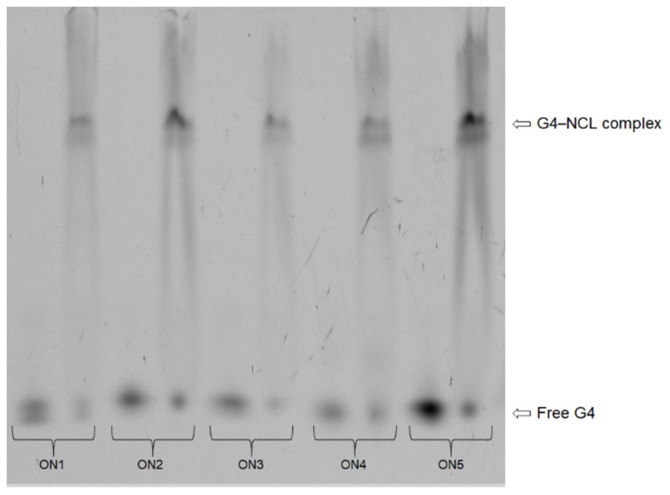
Binding ability of oligonucleotides ON1 to ON5 to interact with nucleolin at a 1:60 ratio. In the EMSA gel, the G4–NCL complexes indicated the ability of each G-quadruplex studied to bind to nucleolin. Reaction mixtures containing only free G-quadruplex.

Herein, we have examined the ability of the analyzed 5′-FAM-labeled oligonucleotides (ON1 to ON5) to bind to nucleolin to verify whether the observed inhibitory effects on *HeLa* cells were exerted via a common mechanism assuming the interactions of G-quadruplexes with NCL. The protein binding patterns of oligonucleotides incubated with nucleolin were studied by the electrophoretic mobility shift assay (EMSA), and the resultant data are presented in Figure 7. The analysis of the EMSA results revealed that all the analyzed aptamers could bind to NCL with different levels of efficiency. The most favorable binding parameters were obtained for oligonucleotides ON2 and ON5. Both are characterized by the presence of a core built up with three G-tetrads and long loops containing four thymidines. Additionally, ON5 has an unpaired guanosine residue at its 3′-terminus. In particular, the compounds also possessed beneficial inhibitory activity on *HeLa* cells, likely due to their interactions with nucleolin. Slightly less efficient binding (the difference was statistically non-significant, *p* > 0.05) was observed for ON1, which comprises four G-tetrads and 4-nt-long loops, but this compound exerted no antiproliferative effect. Contrary to the above, ON3, with good inhibitory activity, had an almost up to 30% lower ability to bind NCL (Table 3, the difference was statistically significant, *p* ≤ 0.05). The structure of this oligonucleotide comprises three G-tetrads, long loops containing four thymidines and an unpaired guanosine at the 5′-terminus. The lowest value of binding was achieved for ON4, formed by four G-tetrads and 3-nt-long loops. The presented findings suggest that the most beneficial factor for interactions with nucleolin is the presence of a longer loop, and that decreasing the nucleotide number in this region can result in reduced binding ability. Our experiments are consistent with previously published results [45]. Teulade-Fichou et al. have demonstrated that NCL binds to the G-quadruplexes via, among other elements, single-stranded central loops and that the affinity of these interactions strongly depends on the length of this G4 fragment. The only exception to the above rule constitutes ON3. Although ON3 possesses three G-tetrads and a long loop containing four thymidines, its binding affinity was relatively low compared with that of structurally similar compounds. The cause may be the presence of an additional guanosine residue at the 5′-terminus of ON3 and the formation of stacking interactions of this nucleotide with guanosine from the second G-quadruplex-forming strand (Figure 1). This presumably might interfere with the binding of ON3 to NCL. Importantly, although all studied oligonucleotides show an ability to bind with nucleolin, it is not possible to exclude that the ON1–ON5 can act via other nucleolin-independent mechanisms.

## 3. Materials and Methods

### 3.1. Chemical Synthesis of Oligonucleotides

The oligonucleotides listed in Table 1 were synthesized on an automated RNA/DNA synthesizer using the standard phosphoramidite approach with commercially available phosphoramidite building blocks. The deprotection steps were performed according to previously used and described protocols [32,48]. The composition of all oligonucleotides was confirmed by MALDI-TOF (Bruker Autoflex, Billerica, MA, USA) mass spectrometry.

### 3.2. UV Melting Studies

The single-stranded oligonucleotide concentrations were calculated based on their absorbance at 85 °C, and the extinction coefficients were calculated using the OligoAnalyzer tool (Integrated DNA Technologies). UV melting analysis was performed for nine different concentrations of each oligonucleotide in the range of 10^−4^ to 10^−6^ M. The oligonucleotides were dissolved in buffer containing 100 mM potassium chloride (KCl), 20 mM sodium cacodylate and 0.5 mM Na_2_EDTA (pH 7.0). The buffer was degassed at an elevated temperature before the measurements. Absorbance versus temperature curves were obtained using the UV melting method at 295 nm with the temperature range of 95 to 3 °C and a temperature decrease of 0.2 °C/min (Supplementary Data, Figure S9) using a JASCO V-650 (Cremella (LC) Italy) spectrophotometer equipped with a thermoprogrammer. The thermodynamic parameters were analyzed and determined using MeltWin 3.5 software. The melting temperatures calculated for the 10^−4^ M concentration of the oligonucleotide are denoted by T_M_, and the melting points for any other concentration of oligonucleotide are denoted by T_m_.

### 3.3. Circular Dichroism Spectra

The measurements of CD signals were performed using the JASCO J-815 (Cremella (LC) Italy) spectropolarimeter. G-quadruplex oligonucleotides were dissolved in buffer containing 100 mM KCl, 20 mM sodium cacodylate and 0.5 mM Na_2_EDTA (pH 7.0) to reach a sample concentration of 3.0 μM. The G-quadruplex samples were denatured at 90 °C for 3 min and then were gradually cooled to room temperature overnight, followed by data collection. The spectra were recorded in triplicate at 37 °C in the 210–320 nm wavelength range. Data analysis was performed using Origin v8.5 software.

### 3.4. Thermal Difference Spectra

G-quadruplex oligonucleotides were dissolved in buffer containing 100 mM KCl, 20 mM sodium cacodylate and 0.5 mM Na_2_EDTA (pH 7.0) to achieve a sample concentration of 3.0 μM. The G-quadruplex samples were denatured at 90 °C for 3 min and then were gradually cooled to room temperature overnight, prior to data collection. The TDS measurements were performed using a JASCO V-650 (Cremella (LC) Italy) spectrophotometer equipped with a thermoprogrammer. The absorbance spectra were collected in triplicate at 4 and 90 °C in the 220–335 nm wavelength range. Thermal difference spectra were obtained by subtraction of the low-temperature from the high-temperature absorbance spectrum. Origin 8.5 software was used for spectral analysis. The differential spectra were normalized by dividing the data by their maximum values.

### 3.5. Cell Culture

The human cervical adenocarcinoma (*HeLa*) cell line was purchased from American Type Culture Collection (ATCC, Rockville, MD, USA). Cells were cultured in RPMI 1640 medium supplemented with 10% fetal bovine serum (FBS) (Gibco, Waltham, MA, USA), 1% Antibiotic–Antimycotic solution (Gibco, Waltham, MA, USA) and 1% MEM Vitamin solution (Gibco, Waltham, MA, USA). The cells were grown in an incubator at 37 °C with 5% CO_2_ and a relative humidity of 95%.

### 3.6. Antiproliferative Assay

The antiproliferative properties of the oligonucleotides were evaluated using the MTT assay. The G-quadruplexes were dissolved in 1× PBS buffer with 100 mM potassium chloride (KCl) to a final concentration of 10 µM, followed by denaturation at 90 °C for 3 min and then cooling to room temperature overnight. The experiments were performed on *HeLa* cells, which were seeded in 96-well plates at a density of 500 cells/well in 100 μL of RPMI 1640 medium (Gibco, Waltham, MA, USA) supplemented with 10% FBS (Gibco, Waltham, MA, USA) and MEM 1% vitamin solution (Gibco, Waltham, MA, USA). The 96-well plates were incubated at 37 °C, 5% CO_2_ and a relative humidity of 95% for 24 h. After that, *HeLa* cells were exposed to a 10 μM concentration of G-quadruplex oligonucleotides for 7 days. Subsequently, the growth medium was removed and 1x MTT solution (Sigma-Aldrich, Darmstadt, Germany) in RPMI 1640 media was added to the wells. The cells were incubated at 37 °C in 5% CO_2_ and a relative humidity of 95% for 4 h. Next, the medium was removed and replaced with an aqueous combination of 70% isopropanol and 40 mM HCl (100 μL/well) to dissolve the blue-purple crystals of formazan. The plates were shaken at 300 rpm at room temperature for 30 min. The quantity of free formazan was measured at 595 nm using a microplate reader xMark (Bio-Rad, CA, USA). Data analysis was performed using Microsoft Excel 2016 software. Each experiment was repeated in triplicate, and the results are expressed as the means ± SD.

### 3.7. Cellular Uptake 

*HeLa* cells (4 × 10^5^) were seeded in 6-well plates with 2 ml of RPMI 1460 medium (Gibco, Waltham, MA, USA) supplemented with 10% FBS (Gibco, Waltham, MA, USA) and 1% MEM Vitamin solution (Gibco, Waltham, MA, USA) and then were incubated at 37 °C with 5% CO_2_ for 24 h. The 5′-FAM-labeled oligonucleotides (FAM-ONs) were dissolved in 1× PBS buffer with 100 mM potassium chloride (KCl) to a final concentration of 10 µM, followed by denaturation at 90 °C for 3 min and overnight cooling to room temperature. After that, the cells were incubated with oligonucleotide solutions at 37 °C for 2 h. Next, the cells were washed once with ice-cold PBS and then 2 ml of ice-cold DPBS (Gibco, Waltham, MA, USA) containing 1 μg/ml of propidium iodide (PI) was added. The plates were then incubated on ice for 3 min and washed twice with ice-cold DPBS (Gibco, Waltham, MA, USA), followed by the addition of 1x trypsin–EDTA (300 μL) and incubation at room temperature for 3 min. The cells were collected by adding 4 ml of ice-cold RPMI 1640 culture medium (Gibco, Waltham, MA, USA) supplemented with 10% FBS (Gibco, Waltham, MA, USA) and 1% MEM Vitamin solution (Gibco, Waltham, MA, USA). The cell suspension was transferred to 15-ml sterile Falcon tubes and centrifuged at 400× *g* at 4 °C for 5 min. The cell pellet was gently resuspended in 0.5 ml of 1% paraformaldehyde and incubated for 10 min at room temperature. The cells were centrifuged at 400× *g* at 4 °C for 5 min, and the cell pellet was resuspended in 0.5 ml of DPBS (Gibco, Waltham, MA, USA). The solution was transferred to flow cytometer tubes, and the FAM fluorescence was measured using a BD FACS Calibur (Becton Dickinson, NJ, USA) flow cytometer. Ten thousand cells were counted, gated to exclude cell debris and PI labeled (nonviable cells) for analysis. The relative uptake was analyzed by FlowJo v10.6.1 software and determined by comparing histograms and the mean of FAM fluorescence intensity. Each experiment was repeated in triplicate, and the results are expressed as the means ± SD.

### 3.8. Viability of Oligonucleotides in Human Serum

One picomole of each oligonucleotide was dissolved in 20 µl of 1 × PBS containing 100 mM KCl. The samples were denatured at 90 °C for 6 min and cooled overnight to room temperature. Next, 200 µl of human serum from male human AB plasma (Sigma-Aldrich, Germany) was added, and the samples were incubated at 37 °C. Aliquots of 5 µl were removed after 0, 10, 20, 40, 60, 120, 180, 480 and 1440 min of incubation and then were mixed with 5 µl of 70% deionized formamide solution containing 50 mM EDTA, followed by cooling on dry ice to quench the reaction. The samples were loaded on a 12% denaturing polyacrylamide gels prepared in 1× TBE buffer. Denaturing PAGE was performed in 1× TBE buffer at 20 W for 3 h at room temperature. The resultant gel was imaged and quantified by storage phosphor technology using a Fuji PhosphorImager, Fla 5100 (FUJIFILM Life Science, Cambridge, MA, USA) and MultiGauge Analysis Software v3.0. Data analysis was performed using Origin v8.5 software, each experiment was repeated in triplicate and the results are expressed as the means ± SD.

### 3.9. Nucleolin Binding Assay

Human nucleolin was produced as a fragment containing amino acids 284–707 with four RNA-binding domains, the C-terminal RGG boxes and 6 histidines at the C-terminus. Nucleolin expression in *Escherichia coli* was performed using the bacterial pET21a expression vector (Novagen, Madison, WI, USA) containing the encoded nucleolin fragment cloned in the Ndel/Xhol sites (a kind gift from Dr. Leszek Błaszczyk, Institute of Bioorganic Chemistry, Polish Academy of Sciences, Poznań, Poland). The ability of the oligonucleotides to bind nucleolin was determined using the electrophoretic mobility shift assay (EMSA). The 5′ FAM labeled-ONs were dissolved in NCL binding buffer, containing 30 mM sodium phosphate buffer with 100 mM KCl, to a final concentration of 0.25mM, followed by denaturation at 90 °C for 3 min and overnight cooling to room temperature. Binding reactions were conducted by incubating 0.25 mM 5′-FAM-labeled ONs with 15 mM nucleolin in a final volume of 10 µl. Free 5′-FAM-ONs were used as a reaction control. After 30 min of incubation at 37 °C, 5 μL of each binding reaction was loaded onto a 4.5% polyacrylamide native gel (acrylamide:bisacrylamide, 37.5:1 ratio). Electrophoresis was performed at 4 °C for 3 h with constant voltage (200 V) in 1 × TBE electrophoresis buffer. The resultant gel was imaged and quantified by storage phosphor technology using a Fuji PhosphorImager, Fla 5100 (FUJIFILM Life Science, Cambridge, MA, USA) and MultiGauge Analysis Software v3.0. Data analysis was performed using Origin v8.5 software, and each experiment was repeated in triplicate. The results are expressed as the means ± SD.

### 3.10. Statistical Analysis 

The results are reported as the means ± standard deviation, and at least 3 independent biological replicates were performed for the MTT assay, cellular uptake assay and viability assay of oligonucleotides in serum. Data analysis was performed using Sigma Plot software (version 12.5; SysTest Software Inc., El Segundo, CA, USA), and the statistical significance between control and treated cells was tested by one-way ANOVA. Normality was tested by the Shapiro–Wilk test. The differences were considered statistically significant for *p* < 0.001.

## 4. Conclusions

The sequence-related G-quadruplex structures described herein were selected based on their similarity in loop length or the number of G-tetrads in the core. Thermodynamic studies demonstrated that all G-quadruplexes fold intermolecularly with a tendency toward the increased thermodynamic stability of variants possessing more G-tetrads in the core. Moreover, the loop length also influences the stability of the studied G-quadruplexes, indicating the 3-nt-long loop as energetically most preferential for the formation of a specific loop type. Nevertheless, differences in the distribution of electrostatic forces caused by the various widths of G-quadruplex grooves might also contribute to alteration in the thermodynamic stability of G-quadruplexes with the same number of G-tetrads. CD analysis showed that slight changes in the number of G-tetrads or length of loops influence the structure folding, revealing antiparallel, hybrid or mixed topology of the studied G-quadruplexes. As expected, the presence of an additional aromatic system, i.e., a guanosine residue at the 5′- or 3′-terminal position, stabilizes the G-quadruplex structure. By contrast, the presence of fluorescein at the 5′-end causes destabilization of the G-quadruplex structures due to specific structural restrictions. Importantly, all unlabeled G-quadruplexes are stable at physiological temperature. 

The antiproliferative studies revealed that the G-quadruplex inhibitory activity is strongly dependent on its structure. It should be emphasized that although some variation in the results of UV analysis and MTT assay for FAM-labeled and unlabeled ON1–ON5 could be observed, the overall tendency was generally unchanged, therefore we were able to drawn some general conclusions about structure–activity relationships for the analyzed set of oligonucleotides (Supplementary Data, Figure S10). The oligonucleotides with a lower number of G-tetrads in the core and longer loops are more predisposed to act as an effective inhibitor of cancer cell growth. Generally, the above statement is also reflected in the ability of G-quadruplexes to bind nucleolin. Although all the analyzed G-quadruplexes can bind to NCL with different levels of efficiency, the most favorable condition for strong interaction with protein is the presence of a shorter core and 4-nt-long loops. Additionally, the availability of the latter part to the surrounding solution also plays an important role. By contrast, the biostability of the analyzed oligonucleotides and efficiency of their internalization are strictly proportional to their thermodynamic properties, favoring a structuralized and extended G-tetrad core with shorter loops.

The results presented herein clearly outline that the final anticancer activity is a complex, net result of various factors, e.g., the tendency to form a G-quadruplex structure (thermodynamic stability), type of structural motifs, efficiency of cellular uptake, nuclease resistance or ability to bind to cell-surface nucleolin. The optimal anticancer agent should be characterized by effective cellular uptake and remarkable antiproliferative activity; however, these properties in the case of G-quadruplex-based drugs possess partially contradicted structural preferences. Thus, only sensible compromises between optimal structural features, which would facilitate effective cellular uptake and relatively efficient decay in the intercellular compartment, can guarantee therapeutic success. Understanding the pivotal requirements of the G-quadruplex structures that influence the final antiproliferative potential can facilitate the reasonable development of G-quadruplexes with superior anticancer properties. 

## Figures and Tables

**Table 1:** Thermodynamic parameters of G-quadruplex formation ^a.^

Name	Sequence (5′–3′)	Number of G-Tetrads	Number of Thymidine Residues in Loop (nt)	T_M_^−1^ vs. log C_T_ plots				
				−ΔH° (kcal/mol)	−ΔS° (eu)	−ΔG°_37_ (kcal/mol)	T_M_ ^b^ (˚C)	*T_M_ ^c^* *(˚C)*
**ON1**	GGGGTTTTGGGG	4	4	36.5 ± 2.1	91.0 ± 6.4	8.30 ± 0.13	61.0	*70.6*
ON2 *	GGGTTTTGGG	3	4	54.9 ± 6.1	158.9 ± 19.7	5.60 ± 0.14	36.6	*44.6*
ON3 *	GGGGTTTTGGG	3	4	47.6 ± 1.0	128.9 ± 3.2	7.62 ± 0.02	50.2	*53.0*
**ON4**	GGGGTTTGGGG	4	3	37.9 ± 1.5	93.5 ± 4.4	8.97 ± 0.09	66.5	*79.6*
**ON5**	GGGTTTTGGGG	3	4	45.9 ± 2.2	128.1 ± 7.1	6.23 ± 0.03	40.8	*56.6*

^a^—Buffer: 100 mM KCl, 20 mM sodium cacodylate, 0.5 mM EDTA(Na)_2_ (pH 7.0), 5′-FAM-labeled oligonucleotides; ^b^—calculated for 10^−4^ M concentration; ^c^—unlabeled oligonucleotides, calculated for 10^−4^ M concentration; *—non-two-state behavior.

**Table 2:** Half-life of the G-quadruplexes.

G-quadruplex	T_1/2_ (min)
ON1	248.03
ON2	24.88
ON3	36.64
ON4	>24 h
ON5	35.34

**Table 3:** Ability of the G-quadruplexes to bind to Nucleolin.

G-quadruplex	Ratio (%)	
	G4–NCL Complex	Free G4
ON1	71.8 ± 5.1	28.2 ± 5.1
ON2	77.3 ± 5.3	22.7 ± 5.3
ON3	56.5 ± 1.5	43.5 ± 1.5
ON4	51.8 ± 6.9	48.2 ± 6.9
ON5	77.3 ± 5.7	22.7 ± 5.7

## Data Availability

All data are presented through the manuscript and Supplementary Materials; no databases were utilized.

## Supplementary Materials

The following are available online at https://www.mdpi.com/article/10.3390/ijms22094941/s1, Figure S1: Tm dependence vs. sample concentration of ON1, Figure S2: Tm dependence vs. sample concentration of ON2, Figure S3: Tm dependence vs. sample concentration of ON3, Figure S4: Tm dependence vs. sample concentration of ON4, Figure S5: Tm dependence vs. sample concentration of ON5, Figure S6: CD spectra at 37 °C of the 5′-FAM-labeled oligonucleotides ON1–ON5, Figure S7: Normalized thermal difference spectra of 5′-FAM-labeled oligonucleotides ON1–ON5, Figure S8: Antiproliferative activity of the FAM-labeled oligonucleotides ON1 to ON5 studied at 10 µM. HeLa cells cultured without oligonucleotides constituted the control, Figure S9: Representative heating and annealing curve of DNA G-quadruplex analyzed with 0.2 °C/min ramp rate, Figure S10: The comparison of general tendency of changes in thermodynamic stability and antiproliferative properties of labeled and unlabeled ON1–ON5.
