# Supplementary material for: G4 Matters—The Influence of G-Quadruplex Structural Elements on the Antiproliferative Properties of G-Rich Oligonucleotides
Source: Int J Mol Sci. 2021 May 6;22(9):4941. doi: 10.3390/ijms22094941 (PMC8125755; doi:10.3390/ijms22094941)
Supplement: Supplementary file 1 [file ijms-22-04941-s001.zip › ijms-1196093-supplementary.pdf]

SUPPLEMENTARY DATA

G4 matters - the influence of G-quadruplex structural elements on antiproliferative properties of G-rich oligonucleotides

Carolina Roxo, Weronika Kotkowiak and Anna Pasternak **

Department of Nucleic Acids Bioengineering, Institute of Bioorganic Chemistry, Polish Academy of Sciences, Noskowskiego 12/14, 61-704 Poznan, Poland;

*To whom correspondence should be addressed. Tel: + 48 618 528 503; Email: apa@ibch.poznan.pl (A.P.); Correspondence may also be addressed to: kawecka@ibch.poznan.pl (W.K.)

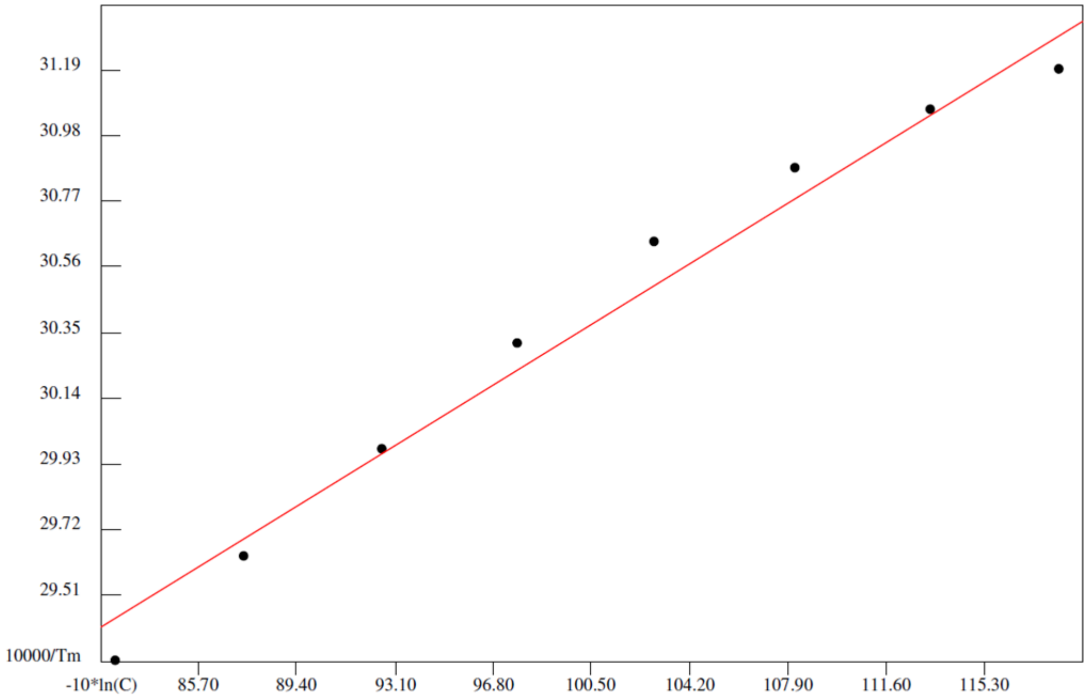

Figure S1. Tm dependence vs sample concentration of ON1.

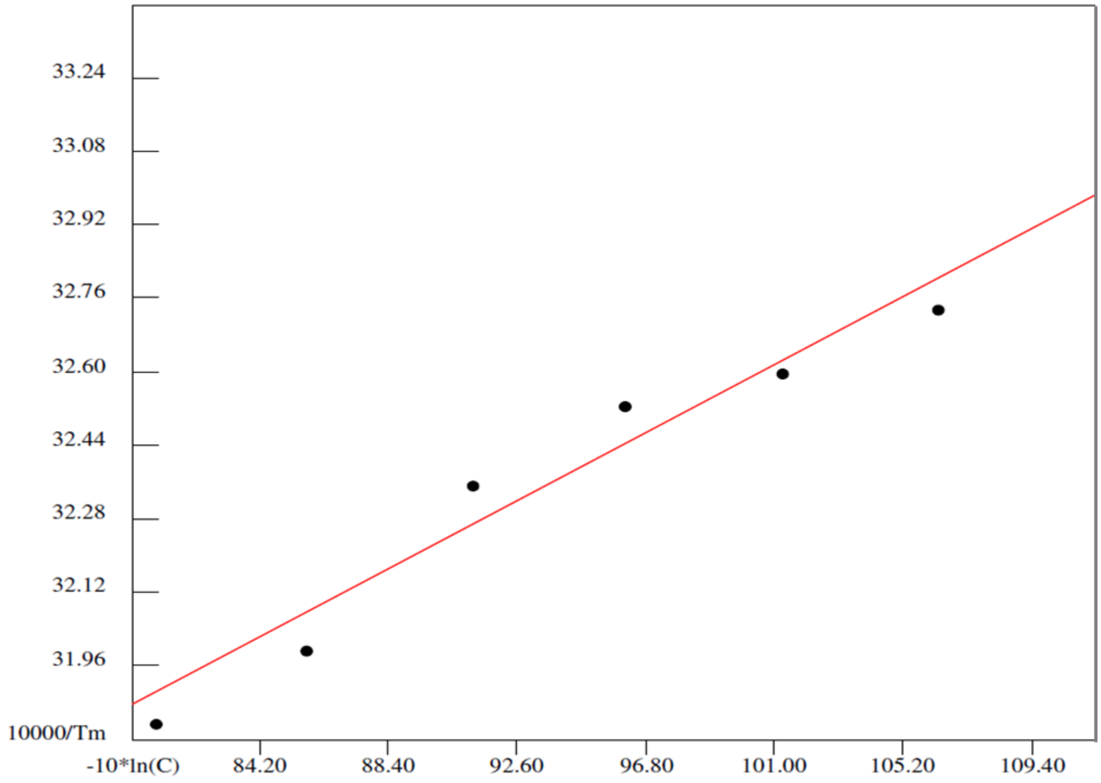

Figure S2. Tm dependence vs sample concentration of ON2.

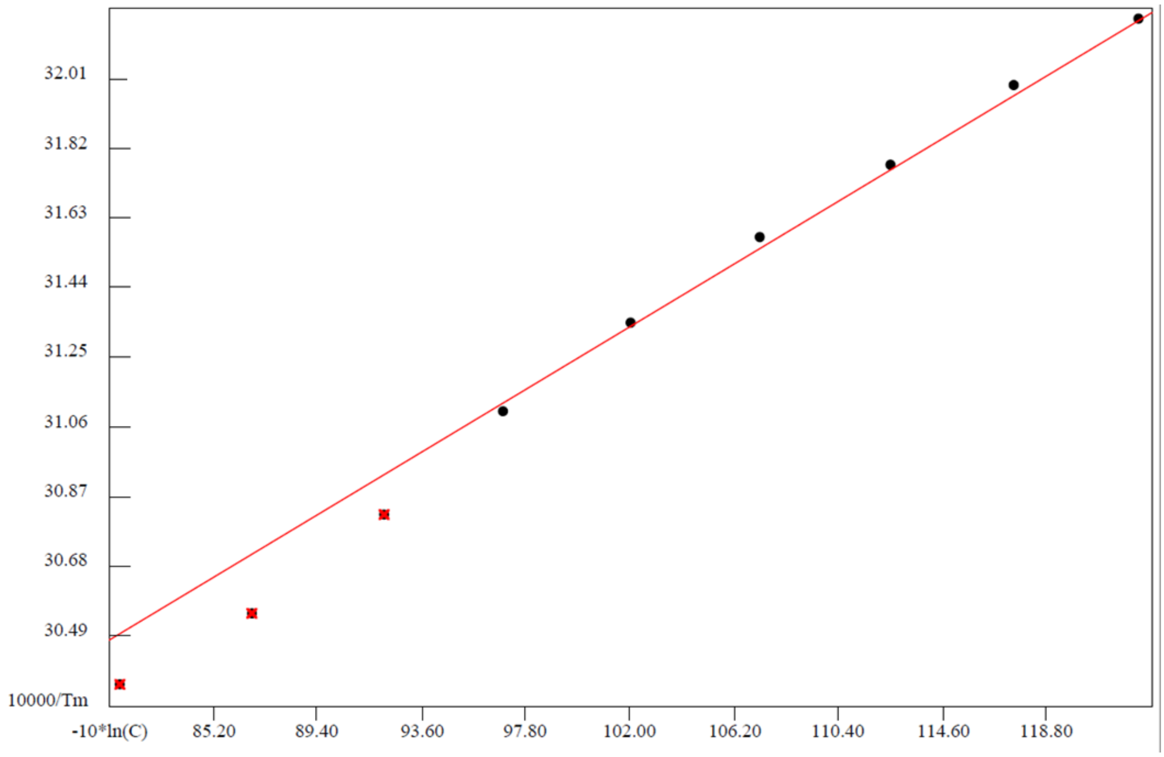

Figure S3. Tm dependence vs sample concentration of ON3.

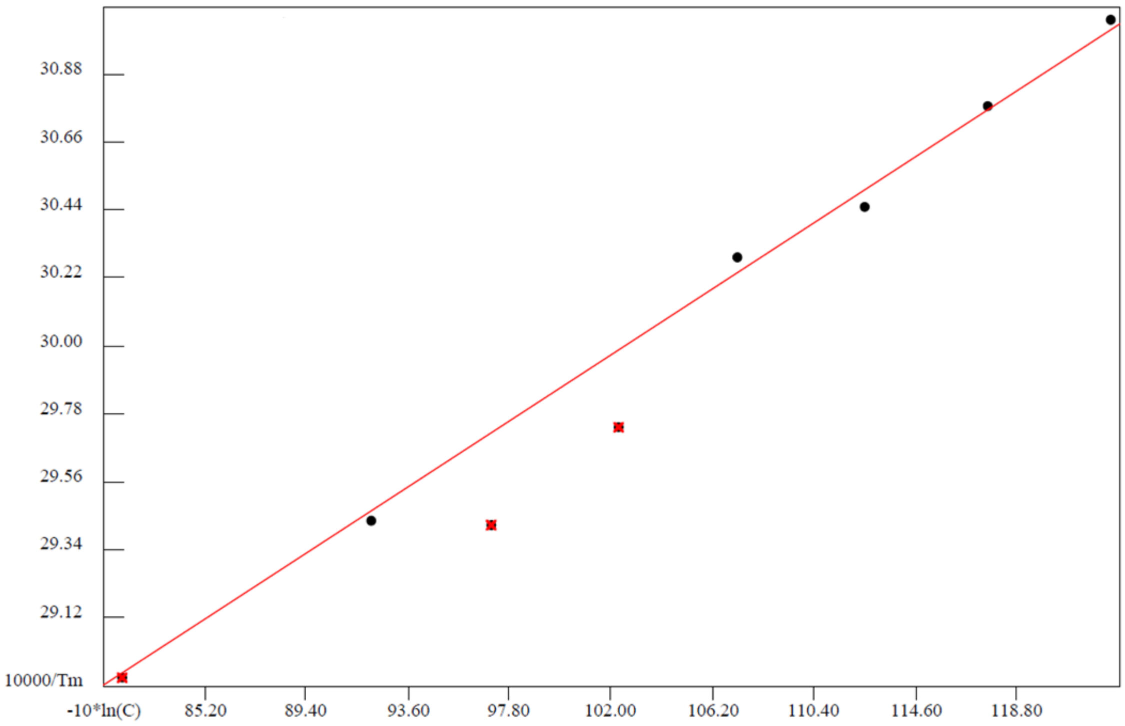

Figure S4. Tm dependence vs sample concentration of ON4.

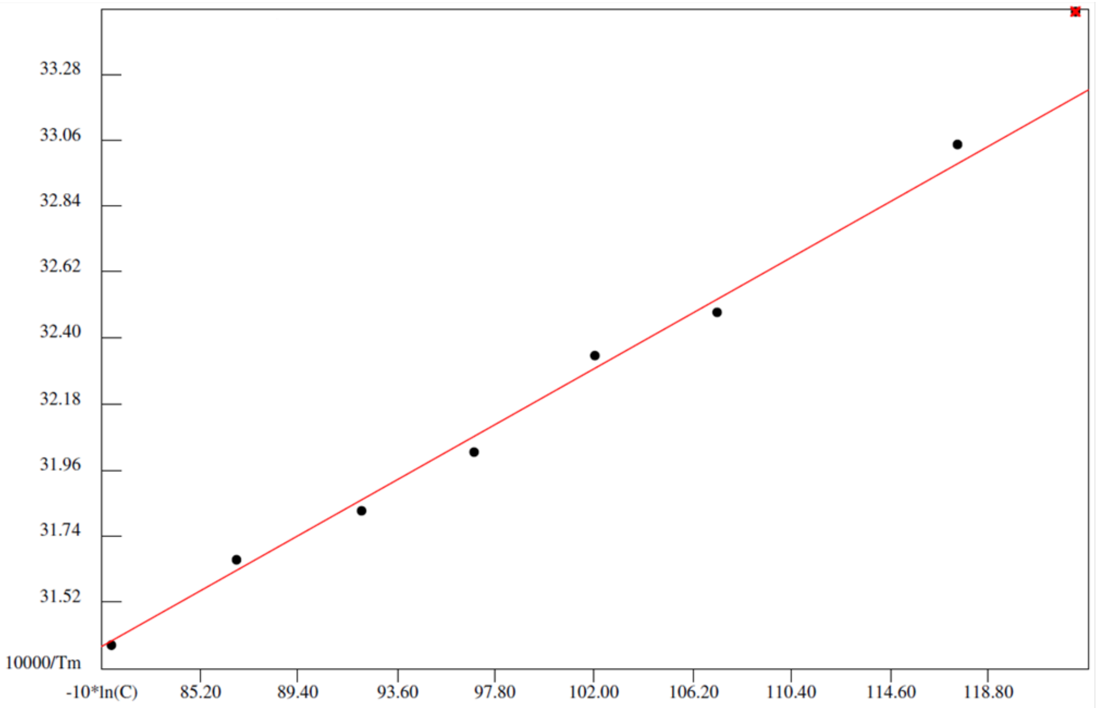

Figure S5. Tm dependence vs sample concentration of ON5.

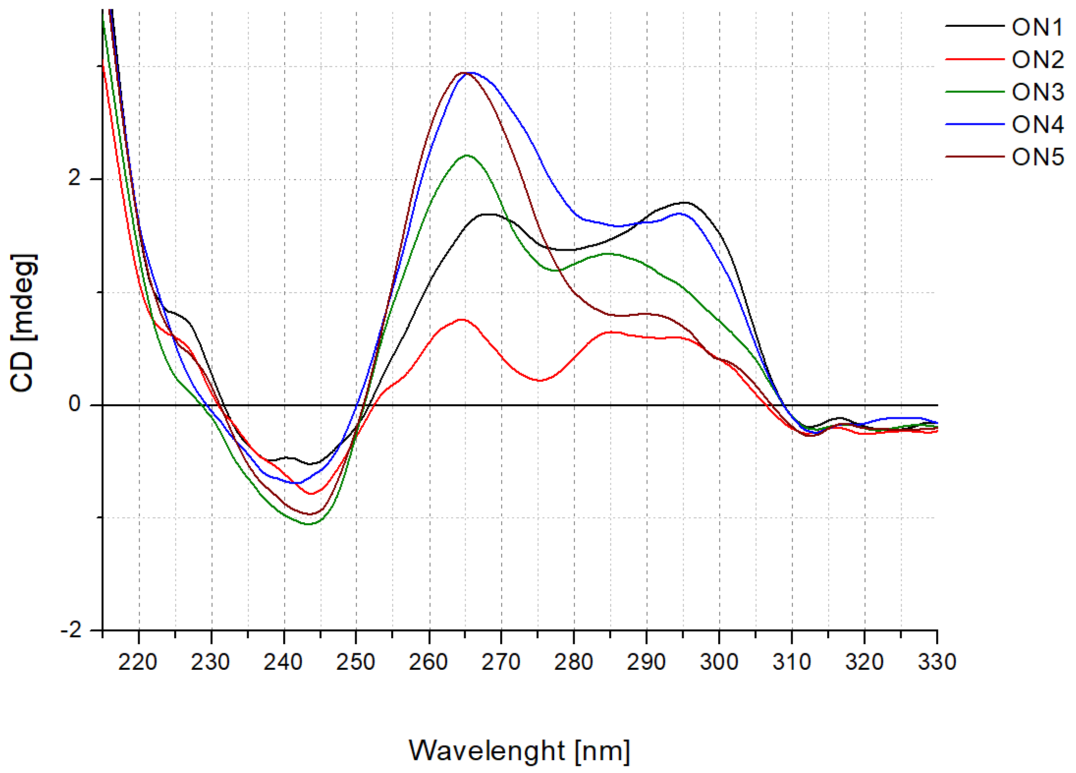

Figure S6. Circular dichroism spectra of 5′-FAM-labeled oligonucleotides ON1 to ON5.

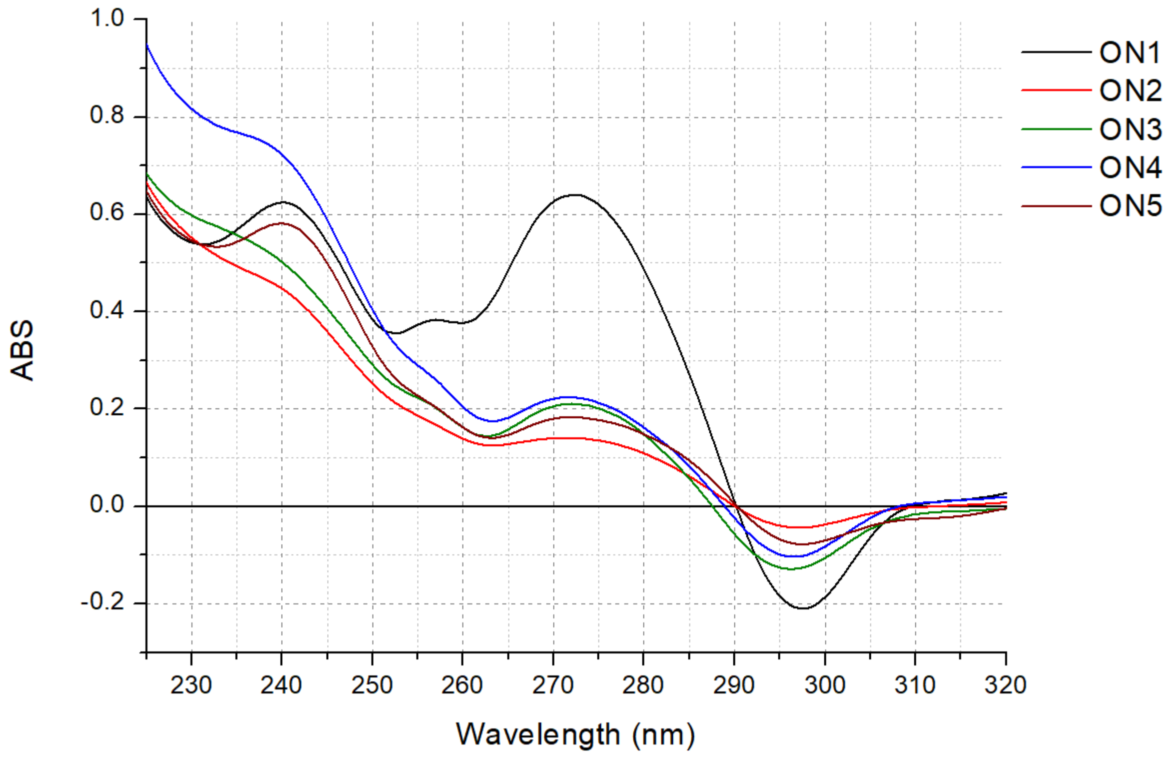

Figure S7. Thermal difference spectra of 5′-FAM-labeled oligonucleotides ON1 to ON5.

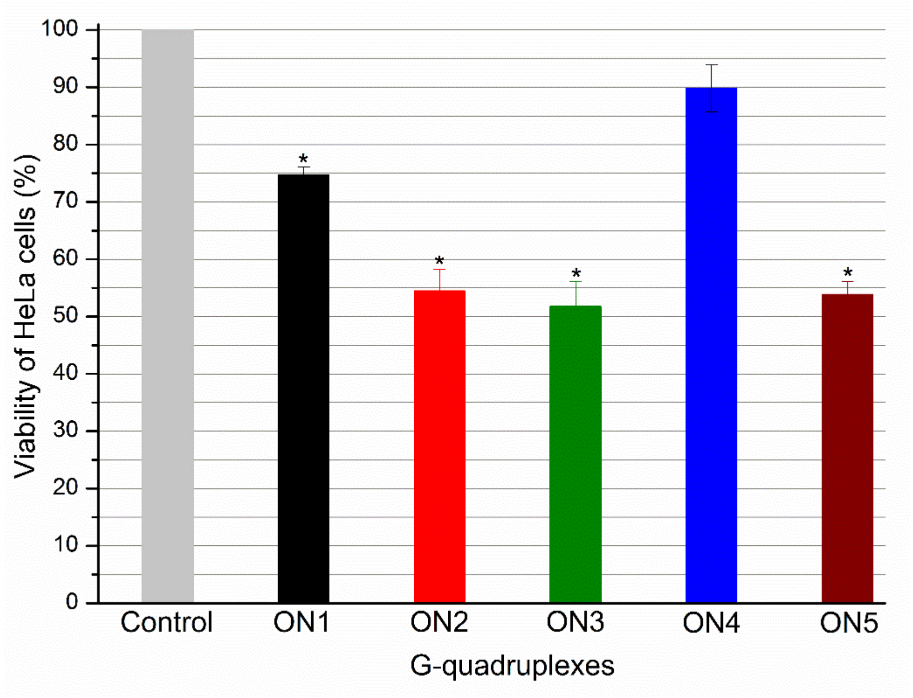

Figure S8. Antiproliferative activity of the FAM-labeled oligonucleotides ON1 to ON5 studied at 10 µM. HeLa cells cultured without oligonucleotides constituted the control. *p<0.001 by unpaired T-test.

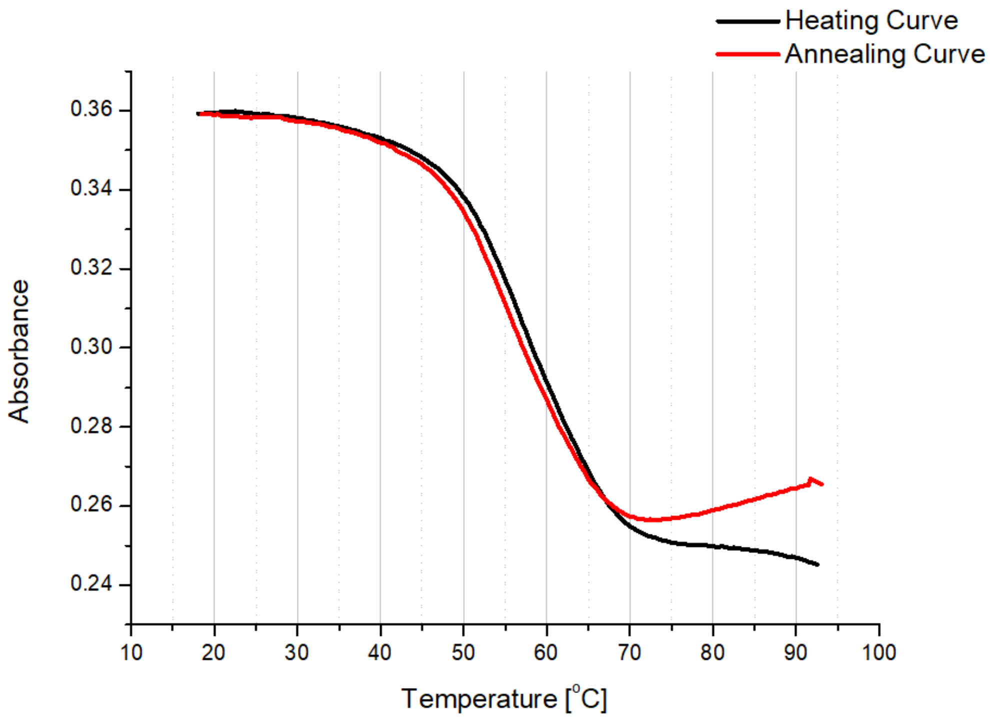

Figure S9. Representative heating and annealing curve for DNA G-quadruplex analyzed with 0.2°C/min ramp rate.

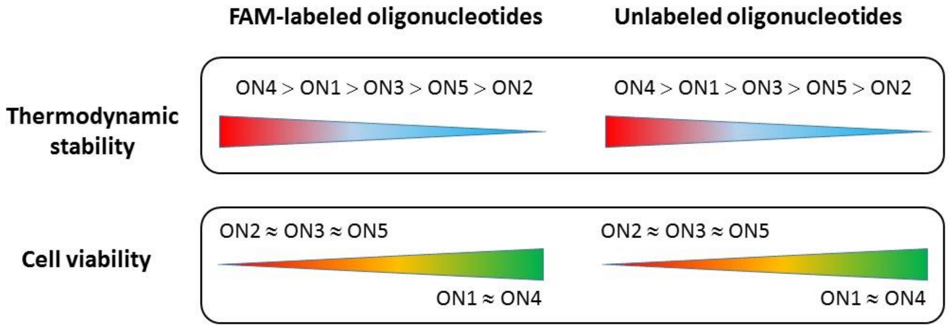

Figure S10. The comparison of general tendency of changes in thermodynamic stability and antiproliferative properties of labeled and unlabeled ON1-ON5.

Table S1. Thermodynamic parameters of G-quadruplex formation.

	Sequence (5'-3')	Average of curve fits				T_M^{-1} vs log C_T plots			
		**-ΔH˚ (kcal/mol)**	**-ΔS˚ (eu)**	**ΔG˚$_{37}$ (kcal/mol)**	**T_M (˚C)**	**-ΔH˚ (kcal/mol)**	**-ΔS˚ (eu)**	**ΔG˚$_{37}$ (kcal/mol)**	**T_M (˚C)**
**ON1***	GGGGTTTTGGGG	66.0± 5.8	174.4 ± 17.3	-11.91 ± 0.65	69.3	45.6 ± 3.9	114.5 ± 11.6	-10.13 ± 0.37	70.6
**ON2**	GGGTTTTGGG	69.4 ± 3.2	199.5± 9.9	-7.49 ± 0.19	45.3	62.5 ± 2.6	177.8 ± 8.3	-7.30 ± 0.06	45.3
**ON3***	GGGGTTTTGGG	70.5 ± 7.0	198.6± 18.8	-8.94 ± 1.22	52.0	37.5 ± 3.1	96.7 ± 9.6	-7.53 ± 0.14	53.1
**ON4***	GGGGTTTGGGG	83.7± 28.7	219.1± 79.3	-15.71 ± 4.19	79.3	40.4 ± 3	96.3 ± 8.8	-10.56 ± 0.34	79.6
**ON5**	GGGTTTTGGGG	64.9± 4.5	178.7± 14	-9.51 ± 0.23	56.4	65.4 ± 5.5	180 ± 16.7	-9.57 ± 0.32	56.6

Buffer: 100 mM KCl, 20 mM sodium cacodylate, 0.5 mM EDTA$(Na)_2$ (pH 7.0), unlabeled oligonucleotides
*-non-two-state behaviour

Table S2. Thermodynamic parameters of G-quadruplex formation.

	Sequence (5'-3')	Average of curve fits				T_M^{-1} vs log C_T plots			
		**-ΔH˚ (kcal/mol)**	**-ΔS˚ (eu)**	**ΔG°_{37} (kcal/mol)**	**T_M (˚C)**	**-ΔH˚ (kcal/mol)**	**-ΔS˚ (eu)**	**ΔG°_{37} (kcal/mol)**	**T_M (˚C)**
**ON1**	GGGGTTTTGGGG	37.3± 3.1	93.3 ± 8.8	-8.39 ± 0.32	61.3	36.5 ± 2.1	91.0 ± 6.4	-8.30 ± 0.13	61.0
**ON2***	GGGTTTTGGG	39.0 ± 3.3	107.4 ± 11.3	-5.68 ± 0.21	37.0	54.9 ± 6.1	158.9 ± 19.7	-5.60 ± 0.14	36.6
**ON3***	GGGGTTTTGGG	61.6 ± 2.1	173.2 ± 5.9	-7.89 ± 0.22	48.6	47.6 ± 1.0	128.9 ± 3.2	-7.62 ± 0.02	50.2
**ON4**	GGGGTTTGGGG	39.6 ± 4.9	98.3 ± 14.6	-9.13 ± 0.4	66.7	37.9 ± 1.5	93.5 ± 4.4	-8.97 ± 0.09	66.5
**ON5**	GGGTTTTGGGG	48.4 ± 3.9	135.8 ± 12.6	-6.28 ± 0.07	40.9	45.9 ± 2.2	128.1 ± 7.1	-6.23 ± 0.03	40.8

Buffer: 100 mM KCl, 20 mM sodium cacodylate, 0.5 mM $EDTA(Na)_2$ (pH 7.0), all oligonucleotides are 5′FAM-labeled
*-non-two-state behaviour
